# Do we need a new CT scan for retreatment of intracranial SRS patients?

**DOI:** 10.1002/acm2.12152

**Published:** 2017-08-03

**Authors:** David Wiant, Matthew Manning, Kyle Koch, Jacqueline Maurer, Lane Hayes, Han Liu, Qingyang Shang, Benjamin Sintay

**Affiliations:** ^1^ Department of Radiation Oncology Cone Health Cancer Center Greensboro NC USA

**Keywords:** repeat CT, salvage, SRS, treatment planning

## Abstract

**Purpose:**

To determine if the treatment planning computed tomography scan (CT) from an initial intracranial stereotactic radiosurgery (SRS) treatment can be used for repeat courses of SRS.

**Methods and materials:**

Twenty‐five patients with 40 brain metastases that received multiple courses of SRS were retrospectively studied. Magnetic resonance scans from repeat SRS (rMR) courses were registered to CT scans from the initial SRS (iCT) and repeat SRS (rCT). The CT scans were then registered to find the displacement of the rMR between iCT and rCT registrations. The distance from each target to proximal skull surface was measured in 16 directions on each CT scan after registration. The mutual information (MI) coefficients from the registration process were used to evaluate image set similarity. Targets and plans from the rCTs were transferred to the iCTs, and doses were recalculated on the iCT for repeat plans. The two dose distributions were compared through 3D gamma analysis.

**Results:**

The magnitude of the mean linear translations from the MR registrations was 0.6 ± 0.3 mm. The mean differences in distance from target to skull on a per target basis were 0.3 ± 0.2 mm. The MI was 0.582 ± 0.042. Registration between a comparison group of 30 CT scans that had the same data resampled and 30 scans that were intercompared with different patients gave MI = 0.721 ± 0.055 and MI = 0.359 ± 0.031, respectively. The mean gamma passing rates were 0.997 ± 0.007 for 1 mm/1% criteria.

**Conclusions:**

The rMR can be aligned to the iCT to accurately define targets. The skull shows minimal change between scans so the iCT can be used for set‐up at repeat treatments. The dosimetry provided by the iCT dose calculation is adequate for repeat SRS. Treatment based on iCT is feasible.

## 1 INTRODUCTION

Up to 40% of cancer patients will develop brain metastases over the course of their illness.^1^ Brain metastases are generally managed with some combination of surgery, whole brain radiotherapy (WBRT), stereotactic radiosurgery, and steroids.^2^ In a radiotherapy setting, SRS offers durable local control with the possibility of reduced neurocognitive impairment compared to WBRT or a combination of WBRT and SRS.^3^ However, the use of SRS alone carries an increased risk of distant brain recurrence versus WBRT, which necessitates active monitoring and follow‐up for patients that have been treated with SRS alone (see 2 and Refs. within). The risk for subsequent metastasis following initial SRS may approach 50%–60%, and these recurrences may be amenable to SRS for salvage.

A typical course of care for a patient treated with linear accelerator based SRS is to undergo magnetic resonance (MR) and a computed tomography (CT) scans prior to an initial treatment with SRS, then to be followed with interval MR imaging. If new metastases are found on review of the interval MR images, the patient returns for a radiotherapy planning CT. After a short period for treatment planning, the patient will undergo salvage SRS treatment.

The primary purposes of radiotherapy planning CTs are to provide information on electron transport for dose calculations and to aid in patient localization during treatment through registration to a cone beam CT (CBCT) or the creation of digitally reconstructed radiographs (DRRs). If these tasks could be fulfilled without the need for a CT, the patient might save the financial cost of the imaging, the radiation dose, and trips to the radiation oncology department associated with a planning CT.

A number of different methods have been used to calculate dose without a CT scan. Treatment planning systems such as GammaPlan for the Gamma Knife (Elekta AB, Stockholm, Sweden) have employed dose calculation models that treated the patient as homogenous media. This system modeled dose to better than 5% accuracy in the absence of air cavities and high density heterogeneities as shown by Monte Carlo simulations.^4^, ^5^, ^6^ In this case, localization was typically provided by stereotactic head frame. Efforts have been made to create “synthetic” or “pseudo” CTs from MR images that can be used for dose calculation and localization.^7^, ^8^, ^9^, ^10^, ^11^, ^12^, ^13^, ^14^ These methods show promise in terms of geometric and dosimetric accuracy, but are not in widespread clinical use at this time.

Brain metastases are difficult to visualize on CBCT or planar x‐ray images used for patient localization in linear accelerator‐based SRS. Because of this, the skull is typically used to align images in the registration process for SRS set‐up. The skull changes little over time, which lends itself to the repeat use of head CTs for SRS set‐up and localization. The intracranial anatomy may change as metastatic disease develops, but changes in the location and electron density of air cavities and high density implants are unlikely. Based on the results of the GammaPlan experience with homogenous dose calculations, small anatomical changes between an initial and repeat CT due to the development of metastatic disease will likely have negligible impact on the SRS dosimetry, which makes the use of repeat CTs feasible with regards to dosimetry.

For image‐guided SRS workflows, the initial CT could be used in lieu of a new CT image with very little change to the workflow. The patient would undergo monitoring MR scans as usual. If a new lesion was detected, the target volumes would be created on the most current MR scan, this MR would be registered to the initial planning CT. Target contours would be transferred to the initial CT and it would be used for dose calculation. The patient would then show up for treatment, without having to come for a new treatment planning CT, where the initial CT would be used for localization. If a thermoplastic mask was used for immobilization at the initial SRS, the same mask would be saved and reused. (New masks could be made as needed.) Any changes in patient orientation from the initial treatment to the repeat treatment would be corrected with 6 degree of freedom positioning device.

The CT simulation impacts the patient and the radiation oncology department on financial and social levels. In terms of the patient, a treatment planning CT may duplicate information in existing diagnostic studies or previous radiation planning CT scans. If prior information could be used, the patient in theory, could be treated on the same day as a follow‐up visit with the radiation oncologist. This would save the patient 1–2 trips (separate treatment planning and treatment delivery visits could be avoided) and multiple hours in the department. Removal of the CT simulation process would also result in a financial savings for the patient. The radiation oncology department would benefit from increased patient throughput and the freedom to plan the treatment without the time pressure associated with a fixed date range between a CT simulation appointment and a treatment delivery appointment. This change in practice may also offer financial benefit to the clinic as we move toward value‐based reimbursement models.

In this work, we evaluate the impact on dosimetry and localization of using an initial CT for repeat SRS planning and delivery.

## 2 METHODS AND MATERIALS

A total of 25 patients that received repeat courses of SRS were retrospectively reviewed. The accuracy of using repeat MR scans (rMR) and initial SRS CT scans (iCT) for target definition and treatment localization at repeat treatments were evaluated. The dosimetric accuracy of using iCTs for dose calculation versus repeat SRS CT scans (rCT) was evaluated.

All the SRS patients were immobilized in Brainlab SRS masks (BrainLAB AG, Feldkirchen, Germany) and underwent CT scans on a Philips Brilliance Big Bore CT scanner (Philips, Cleveland, OH, USA) with a nominal 1 mm slice thickness and a field of view large enough to include the patient and immobilization device. If able, patients received contrast media prior to the CT scan. All patients underwent MR scans prior to the CT scans. The MR scans were acquired on a 3T Signa Excite scanner (GE Healthcare, Waukesha, WI, USA) with 1 mm slice thickness and a field of view large enough to include the patient. Postcontrast T1‐weighted axial scans were used for all cases in this work.

### 2.A Targeting accuracy

Linear accelerator SRS patients are localized using a skull‐based set‐up with a CBCT or DRRs. For set‐up with an iCT at repeat SRS, the skull must be nearly identical in the iCT and rCT to allow for accurate registration of volumetric images or creation of DRRs. We evaluated the similarity of the skull shape, orientation, and the ability to align the skull between the iCT and the rCT using ray traces from each target to the proximal skull surface on each image set. The iCT and rCT were rigidly registered in MIM Maestro (MIM Software, Cleveland, OH, USA). The registration was evaluated and adjusted as needed by a board certified medical physicist. The registered image sets were saved and exported in DICOM format to an offline computer. Here, a custom Matlab (Mathworks, Natick, MA, USA) program was used to evaluate the distance from each target to the proximal skull surface in 16 different directions. The skull surface was identified using a gradient‐based edge detection algorithm. The difference in path length along each ray was used to compare the skull between iCT and rCT.

**Figure 1:**
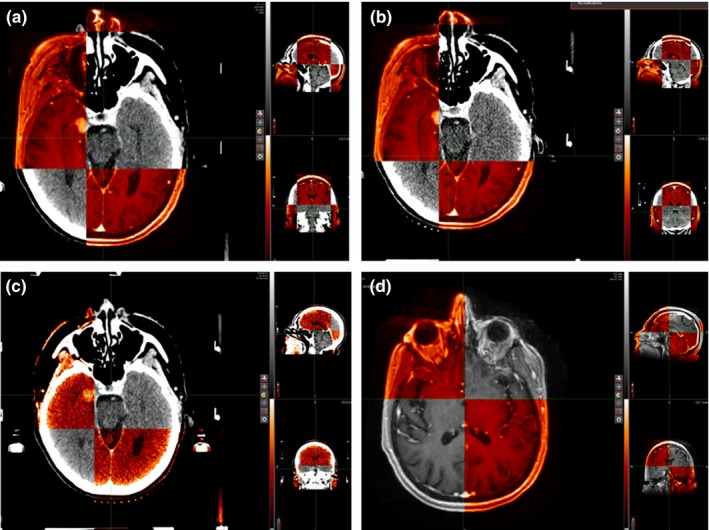
A diagram showing the steps used to compare rMR‐iCT and rMR‐rCT registrations. (a) rMR to rCT registration. (b) rMR to iCT registration. (c) iCT to rCT registration. (d) The rMRs from steps ‘a’ and ‘b’ are registered. Assuming perfect alignment of the iCT to rCT, the differences in shifts from the iCT‐rCT registration and rMR‐rMR registrations are the difference between registering the rMR to iCT and rMR to rCT.

For SRS target definition using an iCT, the intracranial soft tissue targets defined on the rMR must be accurately positioned on the iCT scan relative to the skull, which is based on an iCT to rMR registration. The ability to register the rMR to the iCT was evaluated in a multistep process: (a) the rMR was registered to the rCT using all anatomy, (b) the rMR was registered to the iCT primarily using bony anatomy, (c) the iCT was registered to the rCT, (d) the residual shifts to align the rMR image set from the registrations in steps 1 and 2 were found. Here, we assume that the iCT to rCT registration is perfect, so that the residual shifts to align the rMR image sets represent the uncertainty in the rMR to iCT registration process. All registrations were performed manually by a board certified medical physicist. The registration process, with examples of typical registrations, is outlined in Fig. 1.

### 2.B Dosimetric accuracy

The dosimetric accuracy using a rCT scan depends on the similarity of the information in the rCT as compared to the iCT. The similarity of the iCT and rCT image sets were compared using the mutual information (MI) and Pearson's correlation (PC) coefficients generated by MIM Maestro in the registration process.^15^ The iCT to rCT metrics were compared against groups of SRS patients that (a) underwent multiple CT scans of the brain on the same day, (b) had CT image sets resampled in the Brainlab iPlan treatment planning system with the voxel size unchanged, and (c) consisted of different SRS patients. The comparison groups were immobilized in Brainlab SRS head frames (BrainLAB AG, Feldkirchen, Germany) or custom thermoplastic masks. Five of the patients that received multiple scans on the same day had CBCT scans on a Varian TrueBeam (Varian Medical Systems, Palo Alto, CA, USA) with 2 mm slice thickness. All other patients had CT scans on the Philips system as described above.

The relationships between MI and PC to time between CT scans were evaluated using Pearson's r value. Correlation values of |r| < 0.4 were considered weak, 0.4 < = |r| < = 0.7 intermediate, and |r| > 0.7 strong. Statistical significance was defined as *P* < 0.05.

The dosimetry was evaluated by calculating the same treatment plan on both image sets. The iCT were rigidly registered to the rCT in Varian Eclipse based on the skull. The targets and plans from the rCT were transferred to the iCT. Dose for the repeat plan was recalculated on the iCT. All planning was done in the Varian Eclipse treatment planning system version 13.6. All plans used dynamic conformal arcs to deliver 20 Gy to cover >98% of a single target with each isocenter and the analytical anisotropic algorithm with a 1 mm dose grid. All planning target volumes (PTV) consisted of a gross tumor volume (GTV) defined on the MR plus a 1 mm expansion.

The two dose distributions were compared through 3D gamma analysis.^16^ The gamma analysis was carried out in custom software written in Matlab. To perform the gamma analysis, the doses from the iCT and rCT calculations were exported in DICOM format to an offline computer. Gamma analysis was performed with a 1 mm distance to agreement (DTA) and a 1% dose agreement percentage. The dose evaluations were based on the global maximum dose. The 1 mm^3^ dose grid was resampled to 0.2 mm^3^ for the gamma evaluation. All dose points greater than 10% of the maximum dose were considered.

The relationships between gamma passing rates and time between scans, as well as the relationships between gamma passing rates and target volume were evaluated using Pearson's r value. Correlation strengths are defined as above.

This work was carried out with the approval of the Cone Health institutional review board under protocol number 1741.

## 3 RESULTS

A total of 30 different CT scans were compared from the 25 SRS patients (some patients had rCTs at multiple time points). The mean time between iCT and rCT was 220 ± 189 days (1 STD) (range 65–979 days). Contrast was administered on all MR scans. Contrast was used on both the iCT and rCT in 22 cases, on either the iCT or rCT in six cases, and on neither CT in two cases.

### 3.A Targeting accuracy

**Figure 2:**
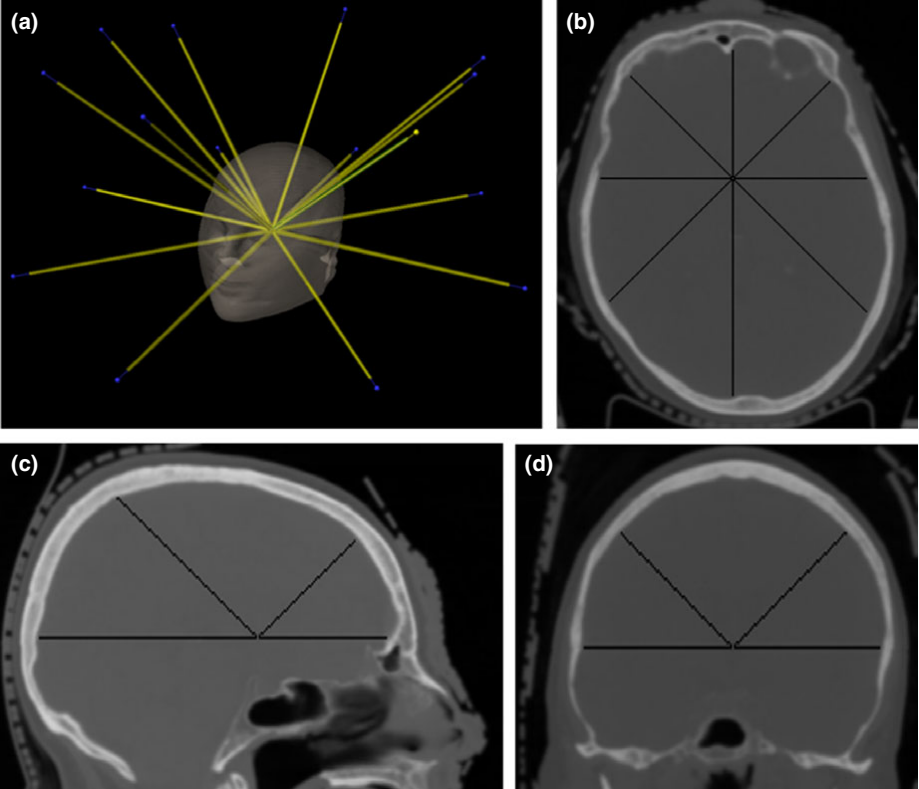
(a) A schematic diagram showing the directions of the rays traces used to measure target to skull distance. The set‐up had eight rays spaced in 45 increments in a transverse plane through the target, and eight additional rays at 45 to the rays in the transverse plane. Examples of the rays in the (b) axial, (c) sagittal, and (d) coronal planes. The solid black lines show the automatically determined distance from target to inner skull surface.

The directions of the ray traces used to evaluate the distance from each target to the proximal skull surface are shown in Fig. 2. The mean absolute difference in distance from target to skull between the iCT and rCT scans over the 640 samples from all patients and all rays was 0.3 ± 0.6 mm (range 0–3.2 mm). The mean absolute difference in distance over all rays for each patient, averaged over all patients, was 0.3 ± 0.2 mm (range 0–0.9 mm).

The magnitude of the residual linear translations over the 30 rMR registrations was 0.6 ± 0.3 mm. Only, six of the cases had displacements >1 mm in magnitude. The full set of mean linear translations and rotations for the rMR image sets are shown in Table 1.

**Table 1:** The residual displacements remaining after the rMR to iCT and rMR to rCT registrations. The numbers in parentheses are the ranges of values

Magnitude (mm)	x (mm)	y (mm)	z (mm)	Roll (°)	Pitch (°)	Yaw (°)
0.6 ± 0.3	0.3 ± 0.2	0.4 ± 0.3	0.3 ± 0.2	0.3 ± 0.2	0.3 ± 0.3	0.3 ± 0.3
(0.0–1.2)	(0.0–1.0)	(0.0–1.1)	(0.0–0.9)	(0.0–0.9)	(0.0–1.0)	(0.0–1.0)

### 3.B Dosimetric accuracy

**Figure 3:**
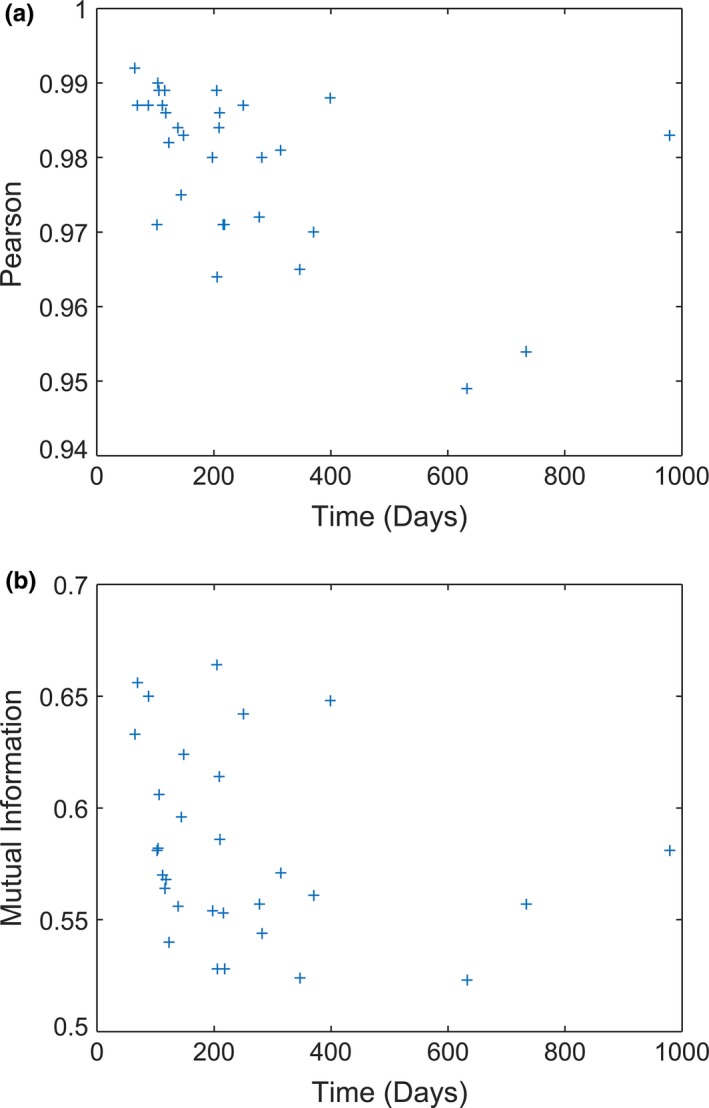
(a) MI and (b) PC as a function of time between CT scans.

The mean MI and PC values for the 30 iCT to rCT comparisons were 0.582 ± 0.042 (range 0.523 – 0.664) and 0.979 ± 0.011 (range 0.949–0.992), respectively. The MI shows no significant correlation with time *P* = 0.18, while the PC shows intermediate, significant correlation with time *r* = −0.51, *P* < 0.01 (Fig. 3).

Registrations between identical image sets gave MI = 1 and PC = 1. A group of 30 SRS patients that had image sets resampled in iPlan had MI = 0.721 ± 0.055 and PC = 0.996 ± 0.002. A group of 30 SRS scans that were compared to scans from different SRS patients had MI = 0.359 ± 0.031 and PC = 0.884 ± 0.032. Comparisons for five patients that each had 2 CBCTs on the same day gave MI = 0.568 ± 0.044 and PC = 0.981 ± 0.011. One patient that underwent three treatment planning CTs on the same day had MI = 0.672 ± 0.026 and PC = 0.994 ± 0.002. The full set of MI and PC values are shown in Table 2.

**Table 2:** The MI and PC values for the different groups of patients

Comparison type	MI	PC
Same patient, same scan	1	1
Same patient, resampled scan	0.721 ± 0.055 (0.649–0.845)	0.996 ± 0.002 (0.989–0.999)
Same patient, different scan on the same day	0.607 ± 0.065 (0.529–0.689)	0.986 ± 0.010 (0.967–0.995)
iCT to rCT (same patient, different day)	0.582 ± 0.042 (0.523–0.664)	0.979 ± 0.012 (0.949–0.992)
Different patients	0.359 ± 0.031 (0.304–0.429)	0.884 ± 0.032 (0.805–0.924)

**Figure 4:**
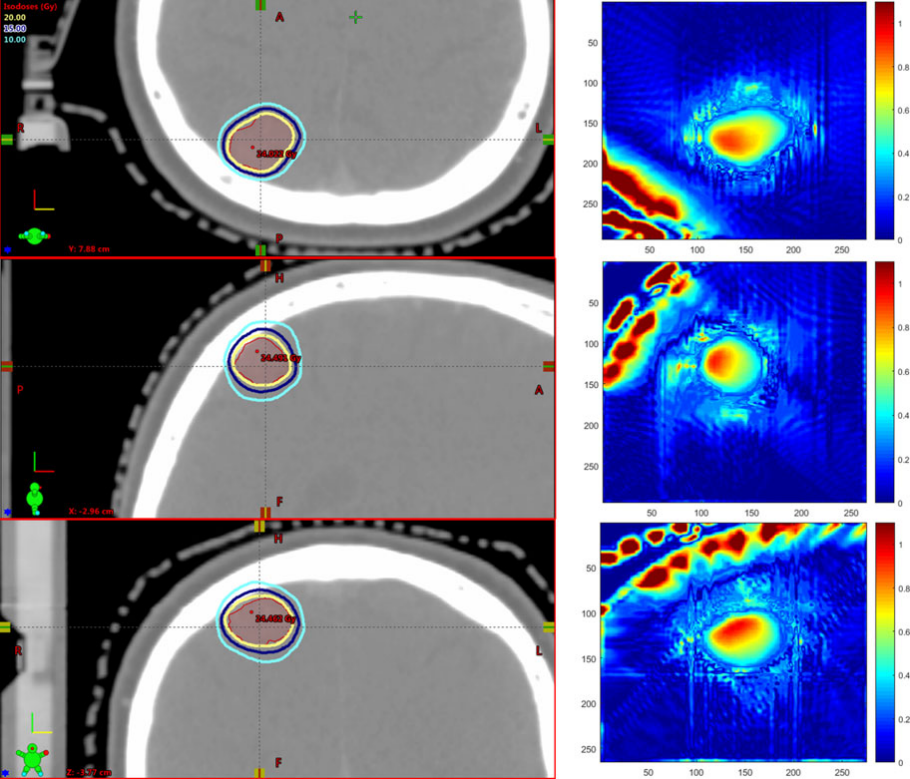
Representative dose distributions and gamma plots. From top to bottom axial, coronal, and sagittal views through the target volume are shown. The target volume is the shaded red region. The inner‐most thick yellow line is the prescription (20 Gy) isodose line. The next thick blue line is the 15 Gy isodose line and the outer‐most thick cyan line is the 10 Gy isodose line. The gamma plots show the points considered in the gamma evaluation (with doses >10% of the maximum dose).

**Figure 5:**
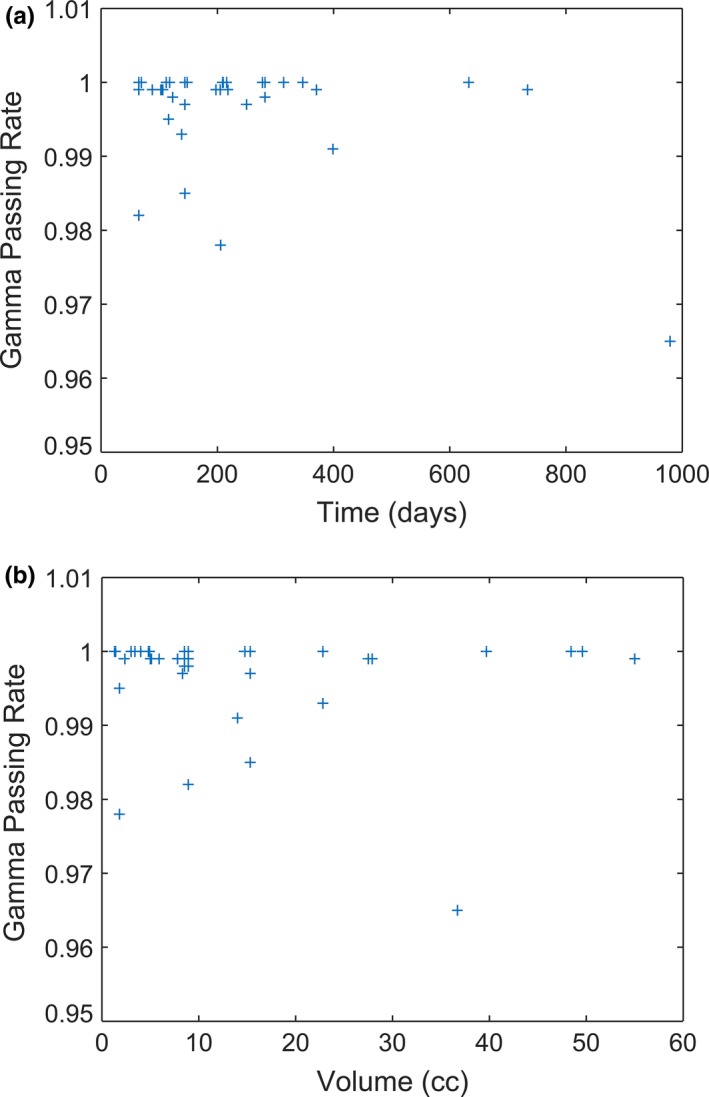
(a) Gamma passing rates as a function of time. (b) Gamma passing rates as a function of volume.

Dosimetry was evaluated for 40 independent targets. The number of targets per patient ranged from 1 to 4. The mean target volume was 1.27 ± 2.09 cm^3^ (range 0.06–10.10 cm^3^). A typical dose distribution and gamma analyses are shown in Fig. 4. The gamma analyses for all patients are shown in Fig. 5. The mean passing rates were 0.997 ± 0.007 (range 0.965–1.000). The gamma evaluations showed no significant correlation with target volume. The gamma evaluations showed moderate, significant correlation with time (*r* = −0.43, *P* = 0.01).

## 4 DISCUSSION

For the iCT to be used for localization at repeat treatments, the skull shape and orientation must be stable and reproducible between the iCT and rCT, so that registrations between CBCT and iCT (or between planar x‐rays and DRRs generated from the iCT) are accurate. The distance from each target to the skull was evaluated in 16 directions for 40 targets over 30 different sets of CT scans. The mean absolute differences in path lengths were on the order of 0.3 mm. The largest mean difference for a single target was 0.9 mm. The largest path length difference for a single ray path length was 3.2 mm (the mean over all path length difference for this target was 0.5 mm).

The typical voxel size for the CT sets used in this work was 0.78 mm × 0.78 mm × 1 mm, which gives a maximum path length across a voxel of 1.49 mm. The mean differences per target are well under 1 voxel. The maximum difference for a single path was on the order of 2 voxels. When the partial volume effects and the subsequent uncertainty in edge detection are considered, <1 voxel difference along target paths indicates minimal difference between the iCT and rCT. Differences between DRRs created from CT and synthetic CTs were found to have similar differences (<1 mm) based on bounding box and landmark analysis.^10^ These differences arise from tissue classification and resampling of the CT information in the synthetic CT creation process. Changes in the iCT to rCT skull are comparable to changes in generating a synthetic CT from a CT. This level of agreement will allow for DRR generation and CBCT registration using the iCT that is suitable for SRS localization.

The magnitude of the residual linear translations from the 30 iMR to rMR alignments was 0.6 ± 0.3 mm. The maximum linear shift was 1.2 mm. All individual rotations were < = 1°. Even the worst case translations were comparable to interoperator variability of 0.4–1.8 mm observed in previous work.^17^, ^18^ Skull‐based registration of the rMR to iCT is capable of accurately positioning targets drawn on the rMR relative to the skull with an uncertainty well within that of image registration, such that there is minimal loss of target definition accuracy relative to the skull using this process. Anecdotally, soft tissue not located near previously treated or newly developed metastatic lesions was stable between scans and was useful for fine tuning MR to CT registrations based on the skull.

Image registration is typically an iterative process without a unique solution that depends on a number of factors that are not directly related to anatomy, such as sampling frequency (and the resulting partial volume effects), patient position relative to the scanner, and scan parameters. Changes in these input parameters between scans may lead to slight variations in MI and PC values when no appreciable anatomic differences exist between the scans. For example, multiple scans acquired within minutes of each other will not be identical due to changes in the image acquisition. Because of these issues MI and PC, admittedly, are not ideal metrics to evaluate anatomic similarity between two specific image sets. However, when considering multiple registrations the increased number of samples helps to reduce the impact of the variable interscan parameters described above and allows MI and PC to give useful information about processes or trends.

Table 2 provides context for the iCT to rCT similarity comparisons. The MI and PC score for the registration of identical image sets is 1. If one of the image sets is resampled through interpolation, while maintaining the same voxel sizes, the mean MI and PC values drop to 0.721 and 0.996, respectively. As described in the last paragraph, starting with identical information and changing the sampling (or the information displayed in each voxel) will affect the image set similarity. The values for the resampled scans set expectations of MI and PC for identical data sets sampled under different conditions.

Comparisons between different patients had mean MI and PC of 0.359 and 0.805, respectively. If we consider just the maximum values from this group of MI = 0.429 and PC = 0.924, all the iCT to rCT values are higher. In all cases, the iCT to rCT image sets are clearly more similar than closely matched intracranial scans from different patients. The MI and PC values from the iCT to rCT are similar to the values from the patients scanned multiple times on the same day, and overlap with the values from the group of patients with resampled scans.

The values for the iCT to rCT registrations are very consistent with scans that were acquired on the same day with minimal anatomic change, and they are much higher than registrations between scans with known anatomic differences, i.e., different patients. Based on the MI and PC metrics, CT scans acquired at different time points for SRS patients are, on the whole, as similar as scans acquired on the same day. This supports the idea that iCT scans will provide similar dosimetry to rCT scans for SRS treatments.

The MI values show no significant correlation with time between the scans. The PC showed a moderate, negative correlation with time between scans. The number of iCT to rCT comparisons separated by more than 400 days is sparse, and the 3 points after 400 days show some fluctuation in PC value. If those points are dropped from the analysis, there is no significant correlation with time. There appears to be no correlation with time for up to 400 days, and there are insufficient data to evaluate beyond 400 days.

The gamma analyses gave a mean agreement of 99.7% with a minimum value of 96.5% over all 40 targets at 1 mm/1% criteria. Any disagreement between the iCT and rCT dose calculations likely arises from a combination of partial volume effects and changes in tissue density between the scans. The resampling of data between multiple CT scans leads to slightly different information contained in each voxel across the scans, particularly at the edges of GTVs and near interfaces due to partial volume effects. Partial volume effects coupled with the finite size of the dose grid and the sharp dose gradients in SRS can yield changes in the dose distribution between the iCT and rCT. Dose changes based on partial volume effects depend on the CT voxel size and appear to be small (<1%) for the voxel sizes used in this work. Changes in intracranial CT number due to contrast and edema variations between scans were found to be on the order of 20 HU in this work. A 20 HU difference over a 20 cm length has been shown to give dosimetric changes of <2% for a 6 MV beam.^19^ The CT number differences between the iCT and rCT are typically local to the target and likely produce dose changes <1%.

The gamma values did show a moderate correlation with time. However, all of the gamma values were high, indicating good agreement between iCT and rCT doses at all times. Also, the lowest gamma score (96.5%) occurred at the greatest time point (979 days). If this point is dropped from the analysis, there is no significant correlation. As described above, there appears to be no correlation with time for up to 400 days, and likely minimal effect beyond 400 days.

We are not aware of other work investigating the use of prior CT images for radiotherapy dose calculations. The most relevant comparison is the use of synthetic CTs to calculate dose for treatment of primary brain tumors. Doses calculated with synthetic CT for conventional treatment of primary brain tumors showed gamma rates of 98%^12^ and 97% (2D analysis through isocenter plane)^9^ at 2 mm/2% criteria and 80% at 1 mm/1% criteria (2D analysis through isocenter plane with a range of 56%–100%).^9^

These studies used 2D gamma analyses to evaluate relatively large and homogeneous (relative to the lower volume, high gradient cases in this work) dose distributions. The 2D gamma analyses should lead to lower passing rates than the 3D method used in this work, while the more homogeneous dose distributions for primary brain tumors should give higher gamma passing rates. Due to these differences, it is not easy to directly compare dose calculations on iCT to these dose calculations on synthetic CTs. However, the comparatively high gamma rates observed in this work strongly suggest that iCT dose calculations are at least as accurate as dose calculations on synthetic CTs for intracranial treatments. With accurate localization, the level of dose agreement found between iCTs and rCTs is certainly adequate for SRS.

The primary treatment concern at repeat treatment would be setting the patient up in the same position as for the initial treatment. This can be easily achieved using the mask from the initial treatment and a 6 degree of freedom positioning (6DOF) device with a CBCT or DRRs created from the iCT. If a new mask is required, care must be taken to maintain consistent pitch (rotation about a left‐right axis) and roll (rotation about a superior‐inferior axis) as most 6DOF devices are limited to 2–3° of rotation. We have reused masks at our institution for the last 5 yr. About 80% of the patients are able to use the mask from the initial course. For the patients that need new masks, some combination of photographs, lasers, and surface imaging should allow the position from the initial mask to be reproduced accurately enough to enable treatment with 6DOF positioning.

The typical workflow at our clinic is to define GTVs on the MR and then to apply a 1 mm expansion to great a PTV. The MR is then registered to the CT for treatment planning. Treatment occurs in <7 days from the acquisition of the MR, so the MR‐based contour is felt to be an adequate. Institutions with other work flows that use CTs for target delineation would need to take this into account when considering iCT treatment for repeat SRS.

Use of the iCT for localization and dose calculation should be carefully evaluated in the presence of surgical intervention between initial and repeat treatments. Changes in intracranial soft tissue due to surgery are not likely to have a marked impact on the dose calculation, as changes in edema were not found to have a big impact on dosimetry. However, the introduction of clips, burr hole covers, or changes to the skull can impact image registrations for target definition and set‐up, and should be carefully considered.

Similar ideas could be applied to other disease sites. For example, palliative bone metastases might be planned based on MR or diagnostic CT scans with acceptable dosimetric and localization accuracy. Further study is planned in this area.

## 5 CONCLUSIONS

The use of iCT and rMRs for repeat treatment planning is feasible and easy to implement. This process allows for target delineation, dose calculation, and set‐up that is adequate for SRS. It may offer time and cost savings to both the patient and the clinic.

## CONFLICT OF INTEREST

The authors declare no conflict of interest.
